# Identification of three distinct cell populations for urate excretion in human kidneys

**DOI:** 10.1186/s12576-023-00894-0

**Published:** 2024-01-02

**Authors:** Yoshihiko M. Sakaguchi, Pattama Wiriyasermkul, Masaya Matsubayashi, Masaki Miyasaka, Nau Sakaguchi, Yoshiki Sahara, Minoru Takasato, Kaoru Kinugawa, Kazuma Sugie, Masahiro Eriguchi, Kazuhiko Tsuruya, Hiroki Kuniyasu, Shushi Nagamori, Eiichiro Mori

**Affiliations:** 1 Department of Future Basic Medicine, Nara Medical University, Kashihara, Nara Japan; 2 Center for SI Medical Research, The Jikei University School of Medicine, Tokyo, Japan; 3 Department of Laboratory Medicine, The Jikei University School of Medicine, Tokyo, Japan; 4 Biological Research Department, Research Institute, Fuji Yakuhin Co., Ltd., Saitama, Japan; 5 RIKEN Center for Biosystems Dynamics Research, Kobe, Hyogo Japan; 6 Graduate School of Biostudies, Kyoto University, Kyoto, Japan; 7 Department of Neurology, Nara Medical University, Kashihara, Nara Japan; 8 Department of Nephrology, Nara Medical University, Kashihara, Nara Japan; 9 Department of Molecular Pathology, Nara Medical University, Kashihara, Nara Japan; 10 Department of Collaborative Research for Bio-Molecular Dynamics, Nara Medical University, Kashihara, Nara Japan; 11 V-iCliniX Laboratory, Nara Medical University, Kashihara, Nara Japan; 12 Present Address: Department of Biological Chemistry and Food Sciences, Faculty of Agriculture, Iwate University, Morioka, Iwate Japan

**Keywords:** Transporter, Single nucleus RNA-sequencing, Uric acid, Human kidney, Gout, Solute carriers

## Abstract

In humans, uric acid is an end-product of purine metabolism. Urate excretion from the human kidney is tightly regulated by reabsorption and secretion. At least eleven genes have been identified as human renal urate transporters. However, it remains unclear whether all renal tubular cells express the same set of urate transporters. Here, we show renal tubular cells are divided into three distinct cell populations for urate handling. Analysis of healthy human kidneys at single-cell resolution revealed that not all tubular cells expressed the same set of urate transporters. Only 32% of tubular cells were related to both reabsorption and secretion, while the remaining tubular cells were related to either reabsorption or secretion at 5% and 63%, respectively. These results provide physiological insight into the molecular function of the transporters and renal urate handling on single-cell units. Our findings suggest that three different cell populations cooperate to regulate urate excretion from the human kidney, and our proposed framework is a step forward in broadening the view from the molecular to the cellular level of transport capacity.

**Supplementary Information:**

The online version contains supplementary material available at 10.1186/s12576-023-00894-0.

## Background

Excretion, the process of biological waste removal, is a vital homeostatic mechanism in all organisms. Humans have turnovers of 3.3 × 10^11^ cells per day [1], and wastes of nucleobases, the basic components of DNA and RNA, are removed with each turnover. Nucleobases are classified into pyrimidine and purine bases. Pyrimidine bases are catabolized to water, carbon dioxide, and ammonia, while purine bases are catabolized to uric acid as a final metabolic waste. Physiologically, more than 90% of serum uric acid exists as monosodium urate, excreted by urate transporters to maintain nucleic acid homeostasis. In other mammals, uric acid is hydrolyzed by uricase (urate oxidase). However, the loss of uricase during primate evolution [2] resulted in urate excretion becoming a more important nucleic acid homeostatic mechanism in humans than in other mammals.

Approximately two-thirds of urate excretion in humans is renal excretion, and the remaining one-third is extra-renal excretion, such as intestinal excretion [3]. Renal excretion in humans is a reabsorption-dominant system composed of reabsorption and secretion [4]. Identification of urate post-secretory reabsorption site [5] led to these physiological urate excretion dynamics being proposed as a “four-component model” [6]. These dynamics are tightly regulated by functional urate transport units (*urate transportome*), which are composed of urate transporters and scaffold proteins [7, 8].

Development of molecular biology has identified at least eleven human genes coding ATP-binding cassette (ABC) transporters or solute carrier (SLC) transporters as renal urate transporters [8–12]. Specific counterparts between influx and efflux transporters are involved in urate reabsorption or secretion [8, 13, 14]. Recently, some urate reabsorption transporters (SLC22A11, SLC22A12, and SLC22A13) are found not to be co-expressed on the apical membrane [15]. However, it is still unclear whether all renal tubular cells express the same set of urate transporters to govern cellular transport direction, and how the distribution of their cells in human kidneys results in physiological urate excretion.

To clarify whether all renal tubular cells express the same set of urate transporters, we analyzed healthy human kidneys at single-cell resolution. We found that not all the renal tubular cells expressed the same set of urate transporters and identified three distinct cell populations for urate handling. Our findings revealed that the molecular functions of the transporters vary and differ across the expression patterns of *transportome* components on single-cell units. Clarification of transporter patterns facilitates the visualization of transport directions at the cellular level. Based on these results, we developed a physiological model for urate handling on single-cell units to visualize the dynamics of urate excretion in human kidneys with higher resolution.

## Methods

### snRNA-seq data sets

We made no distinction between single-nucleus RNA sequencing (snRNA-seq) or scRNA-seq and treated them as scRNA-seq data sets [15]. We sought for scRNA-seq data sets based on two criteria. First, the data sets must be from kidneys of adult human males. Second, each data set must have been as a control in previous studies. The kidney in GSE118184 was validated as a healthy kidney with a serum creatinine measurement of 1.03 mg/dL [16]. Two kidneys in GSE131882 were validated as healthy kidneys via H&E images in which no evidence of glomerulosclerosis, interstitial fibrosis, or immune cell infiltrate was found [17]. Consequently, we selected and downloaded these three data sets from a public functional genomics data repository (Additional file 1: Table S1). Based on procedures shown in other studies, we confirmed that the number of data sets in this study is reliable [17, 18]. The gene names in the data set “GSE118184” and “GSE131882” used HGNC gene symbol and Ensemble gene ID, respectively. We unified the gene names in all data sets as HGNC gene symbols by biomaRt [19, 20].

### Quality control of the data sets

Quality control was processed using the Seurat plug-in of R software [21, 22]. The workflow was built according to the tutorial, “Guided tutorial – 2,700 PBMCs” listed on the Seurat website (https://satijalab.org/seurat/vignettes.html). To exclude low-quality and dying cells, we filtered cells out if their mitochondrial gene content was > 5%, because high mitochondrial gene content is reported to be related to low-quality or dying cells. To exclude empty droplets and cell doublets or multiplets, we filtered out cells with gene counts that were less than 200 or more than each threshold. The thresholds of the three data sets were set at 6000, 6000, and 3000, respectively. Finally, the data sets used in this study consisted of three males and a total of 10,080 cells (Additional file 1: Table S1).

### Multiple data set integration and data pre-processing of cell clustering

Data integration and data pre-processing of cell clustering were also performed using the Seurat plug-in of R software. The workflow was set based on the tutorials, “SCTransform”, “Cell Cycle Regression”, and “Integration and Label Transfer—SCTransform” listed on the Seurat website (https://satijalab.org/seurat/vignettes.html). We selected sctransform as a normalization method; it uses regularized negative binomial regression to normalize UMI count data [23]. Variations in technical factors of scRNA-seq data tend to confound the actual biological variations [24, 25]. We utilized the sctransform normalization and data integration by Seurat in the pre-processing stage to reduce more technical factor variations and preserve real biological variations compared to the standard Seurat workflow [21, 23].

We performed the sctransform normalization twice. The first sctransform normalization was performed with the regression of nFeature_RNA and nCount_RNA, because the calculation of cell cycle regression required the values obtained in the first sctransform. Cell cycle regression is a method used to lessen the effects of cell cycle heterogeneity in scRNA-seq data by calculating cell cycle phase scores based on canonical markers [26]. After the calculation of cell cycle scores, a second sctransform normalization was performed with the regression of nFeature_RNA, nCount_RNA, and cell cycle scores. Finally, we integrated the three data sets according to the tutorial “Integration and Label Transfer—SCTransform” [21].

### Cell clustering and cluster annotation

**Fig. 1:**
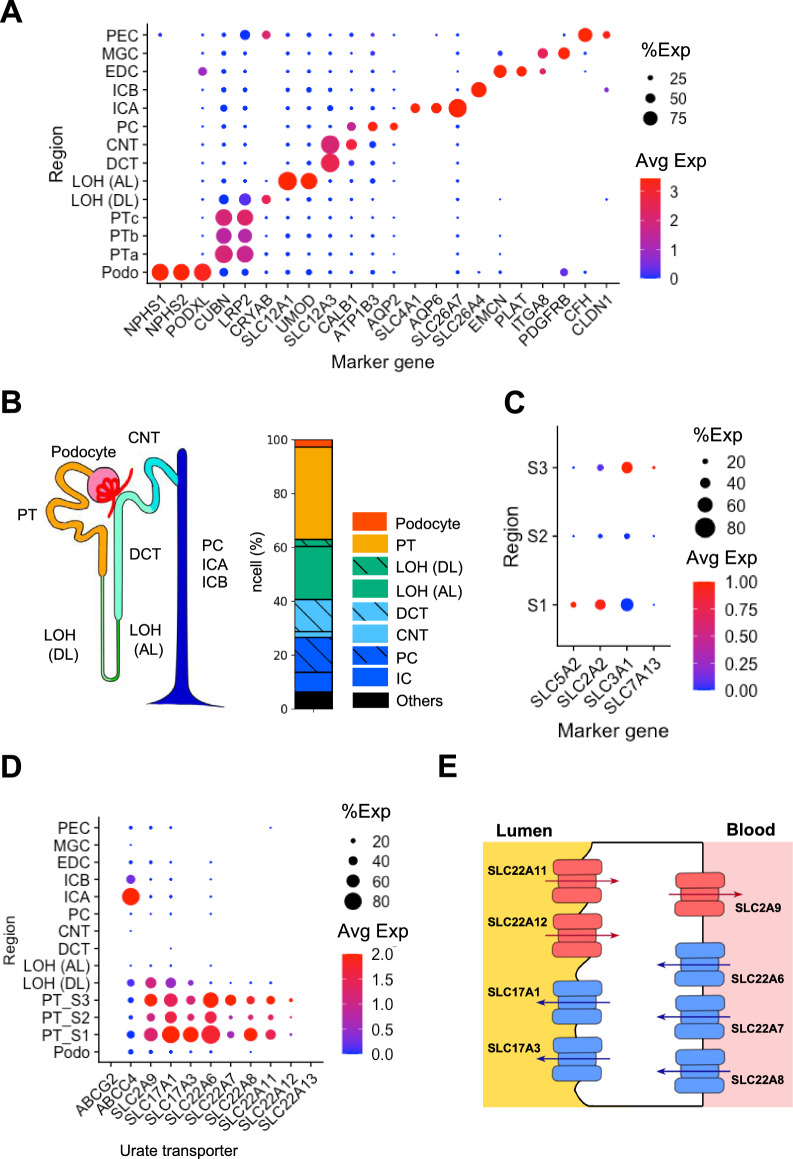
Identification of regions and transporters for urate handling in human kidneys. **A** Annotation of the 14 clusters by the marker genes listed in the Additional file 1: Table S2. Dot plot visualizes how gene expression changes across different clusters (such as anatomical regions, or cell groups). The size of the dot encodes the gene positive percentage (%Exp) of cells within a cluster, while the color encodes the average expression level (Avg Exp) across all cells within a cluster (red is high). Podo: podocyte; PTa-c: three clusters of proximal tubule; LOH (DL): the loop of Henle (descending loop); LOH (AL): the loop of Henle (ascending loop); DCT: distal convoluted tubule; CNT: connecting tubule; PC: principal cell; ICA: intercalated cell type A; ICB: intercalated cell type B; EDC: endothelial cell; MGC: mesangial cell; and PEC: parietal epithelial cell. **B** Left: schematic diagram of human nephron anatomy, and unsupervised clustering of healthy human adult renal cells. Right: bar plot shows proportion of cell clusters of the nephron in all data sets. **C** Dot plot of PT marker genes listed in the Additional file 1: Tables S3: S1 markers and S3 markers. S1–S3, S1–S3 segments of proximal tubules. **D** Dot plot of the gene expression of urate transporters in the renal regions. **E** Schematic diagram of urate transporters expressed in the PT and DL clusters. The apical membrane is to the left of the cells and the basolateral membrane is to the right. Red symbols represent transporters which are reconstituted to urate reabsorption. Blue symbols represent transporters which are reconstituted to urate secretion. Arrows indicate directions of urate flow

To visualize the cell types in the three data sets, we performed cell clustering using the Seurat plug-in of R software. The workflow was built according to the tutorial “Integration and Label Transfer—SCTransform” in the Seurat website (https://satijalab.org/seurat/vignettes.html). A summary of our workflow is presented in Additional file 1: Fig. S1A. First, we grouped the cells based on similarity of gene expression within each single cell by applying unsupervised clustering to all single nuclei after integrating the three data sets. Next, the cell clusters were subjected to annotation. Because cells in distinct anatomical regions have unique expressions of marker genes, the cell clusters were annotated by marker genes (Additional file 1: Table S2). The marker genes were selected based on criteria listed in previous studies [16, 17]. After analysis, 14 clusters were obtained, and these clusters are shown in a dot plot with their regional markers (Fig. 1A).

To visualize the cell clustering, we projected these data sets onto two dimensions with uniform manifold approximation and projection (UMAP), a dimension reduction technique. In the UMAP plots, cells with similar gene expression patterns are placed closer together, while cells with different expression patterns are placed farther apart.

### Annotation of segments in proximal tubule (PT) clusters

PT clusters are anatomically divided into three segments: S1 segment of proximal tubule (S1), S2 segment of proximal tubule (S2), and S3 segment of proximal tubule (S3). Thus, we re-annotated PTa-c clusters (from Fig. 1A) into proximal tubular segments by proximal tubular marker genes that were previously reported (Additional file 1: Table S3). Selection of the marker genes for S1 and S3 segments was based on previous studies [27, 28]. As there was no report of specific marker genes for the S2 segment, the cluster which did not fall into S1 or S3 segments in three PT clusters was assigned as the S2 segment. The expression level of these marker genes across three PT clusters are shown in a dot plot (Fig. 1C).

### Gene positivity and gene negativity

Gene expression data were normalized by the “LogNormalize” method on Seurat. First, gene counts for each cell were divided by the total counts for that cell and the values multiplied by 10,000 were set as the default scale factor on Seurat. Then, these were natural-log transformed. After the log transformation, we defined “gene X”-positivity (the presence of gene X) as cells that expressed “gene X” above the cutoff value. By contrast, “gene X”-negativity (the absence of gene X) was defined as cells that expressed “gene X” less than the cutoff value. A value of ‘0.5’ was set as the cutoff value, because the expression levels had a neckline around the value shown in a violin plot. The gene-positive cell numbers were counted after the translation from the quantitative expression data to binary expression data (gene-positive or negative).

**Fig. 2:**
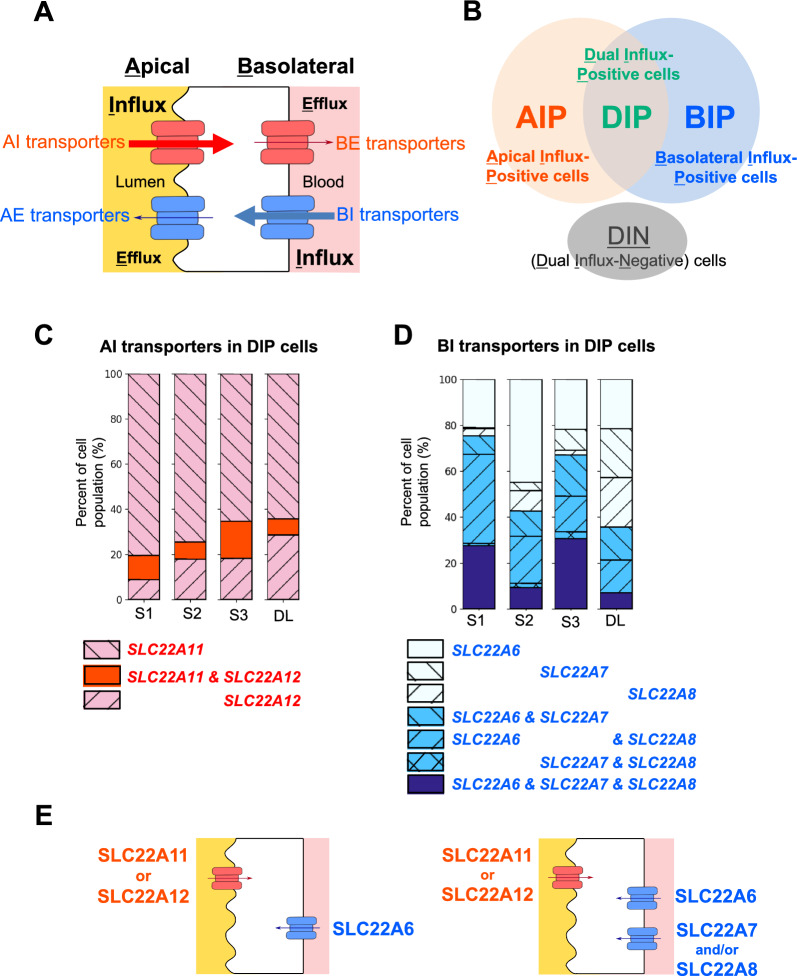
Classification of transporter types and cell populations for urate handling. **A** Schematic diagram of four types of urate transporters classified based on their subcellular localization and physiological roles (apical/basolateral and influx/efflux). The eight transporters were classified into one of the following four transporter types: (1) SLC22A11 and SLC22A12 as Apical Influx (AI) transporters; (2) SLC17A1 and SLC17A3 as Apical Efflux (AE) transporters; (3) SLC22A6, SLC22A7, and SLC22A8 as Basolateral Influx (BI) transporters; or (4) SLC2A9 as Basolateral Efflux (BE) transporter. The cooperation of an AI transporter and a BE transporter leads to urate reabsorption (red). The cooperation of a BI transporter and an AE transporter leads to urate secretion (blue). **B** Venn diagram indicating cell populations, which were classified based on the positivity of influx transporters. Cell clusters in the PT and DL were classified into one of the following cell populations: AI transporter—Positive (AIP) cell population, BI transporter—Positive (BIP) cell population, Dual Influx transporter—Positive (DIP) cell population, or Dual Influx transporter—Negative (DIN) cell population. **C**, **D** Bar plots indicating the percentage (y-axis) of AI transporters (**C**) and BI transporters (**D**) in the DIP cell population across the regions (x-axis) in the DIP cell population. **E** Scheme of expression pattern of influx transporters. The apical membrane is to the left of the cells and the basolateral membrane is to the right of the cells. Red symbols represent the AI transporters, which are only single kind expressed in single cells. Blue symbols represent the BI transporters, which are single or multiple kind(s) expressed in single cells. Arrows indicate directions of urate flow

All urate transporter genes were classified into one of the four types: Apical Influx (AI) transporter, Apical Efflux (AE) transporter, Basolateral Influx (BI) transporter, or Basolateral Efflux (BE) transporter, based on apical/basolateral localization and transport direction as published in the previous study (Fig. 2A, Table 1) [8]. The positivity or negativity of each gene was then examined as described above. Their expression profiles were subsequently constructed into cell populations based on the positivity/negativity of the influx transporters (AI and BI transporters). We classified cell populations into four cell populations: (1) Apical Influx transporter—Positive (AIP) cell population, (2) Basolateral Influx transporter—Positive (BIP) cell population, (3) Dual Influx transporters—Positive (DIP) cell population, or (4) Dual Influx transporters—Negative (DIN) cell population (also shown in Fig. 2B). The AIP cell population had the positivity of at least one AI transporter (*SLC22A11* and/or *SLC22A12*) and the negativity of BI transporters (*SLC22A6*, *SLC22A7*, and *SLC22A8*) (Fig. 2B: AIP). The BIP cell population had the positivity of at least one BI transporter (*SLC22A6*, *SLC22A7*, and/or *SLC22A8*) and the negativity of AI transporters (*SLC22A11* and *SLC22A12*) (Fig. 2B: BIP). The DIP cell population had the positivity of both AI transporters (*SLC22A11* and/or *SLC22A12*) and BI transporters (*SLC22A6*, *SLC22A7,* and/or *SLC22A8*) (Fig. 2B: DIP). The DIN cell population had the negativity of both AI and BI transporters (Fig. 2B: DIN). After that, we subcategorized each cell population by adding the positivity/negativity of efflux transporters (AE and BE transporters) in the same way to define cellular transport directions. Finally, we calculated the cell number presented in Additional file 1: Table S4 as the number of cells which show the positivity of indicated transporters.

**Table 1:** Transport properties of urate transporters expressed in renal proximal tubular regions of human males in this analysis

SLC name	Protein name	Function	Type	Transport system*	Substrates*	Av. (PT_S1)	Av. (PT_S2)	Av. (PT_S3)	Av. (LOH (DL))	Km for urate (μM)	Refs.
SLC22A11	OAT4	Reabsorption	AI	Exchanger/Facilitate	Organic anions (e.g., NSAIDs, PG, urate)	2.99	3.97	4.52	1.94	3779.6	Hagos et al., 2007
SLC22A12	URAT1	Reabsorption	AI	Exchanger	Urate	0.36	0.92	1.42	0.70	371	Enomoto et al., 2002
SLC2A9	GLUT9	Reabsorption	BE	Facilitate	Urate (glucose, fructose)	4.68	4.53	7.35	5.47	365	Anzai et al., 2008
SLC22A6	OAT1	Secretion	BI	Exchanger	Organic anions (e.g., PAH, PG, urate)	10.25	12.93	12.50	1.50	943	Ichida et al., 2003
SLC22A7	OAT2	Secretion	BI	Exchanger/Facilitate	Organic anions (e.g., PAH, PG, urate)	1.29	1.91	4.74	0.79	1168	Sato et al., 2010
SLC22A8	OAT3	Secretion	BI	Exchanger	Organic anions (e.g., PAH, PG, urate)	3.75	2.96	3.13	1.25	2888	Bakhiya et al., 2003
SLC17A1	NPT1	Secretion	AE	Facilitate (Δψ-driven/Cl^−^-dependent)	Organic anions (e.g., PAH, urate)	10.60	8.54	7.66	5.89	1100	Iharada et al., 2010
SLC17A3	NPT4	Secretion	AE	Facilitate (Δψ-driven)	Organic anions (e.g., PAH, urate)	6.02	3.82	3.56	2.18	Unknown	Jutabha et al., 2010

Av.: average expression of three functional cell groups, ITR cell group, DP cell group, and ITS cell group, in each cluster

^*^: a reference to the SLC table (http://slc.bioparadigms.org/)

Column “Ref” shows the reference lists to refer the K_m_ values for urate of each urate transporters

### Visualization of the expression data—dot plots, violin plots, and bar plots

The visualization of the gene expression data was performed using Seurat plug-in of R software [21, 22], ggplot2 plug-in of R software [29], and matplotlib.plt library of Python software [30]. All expression levels used in data visualization were the scaled data after the log transformation, based on procedures described in the previous section. Dot plots of gene expression in each cluster were created by Seurat function, *“Dotplot”*, plug-in of R software. Dot plots indicate how gene expression changes across different clusters (such as anatomical regions or cell groups). The size of the dot encodes the gene-positive percentage (%Exp) of cells within a cluster, while the color encodes the average expression level (Avg Exp) across all cells within a cluster. In other words, the size of the dot indicates the frequency of gene expression, while the color of the dot indicates the level of gene expression across the clusters. Bar plots were created using matplotlib, which is a Python library for producing plots and data visualizations. Plots reside within a *Figure* object created by a function named *“Figure”* in matplotlib.plt library, and the bar plots were made by the *show* method in the *Figure* object.

## Results

### Urate handling transporter identification and localization 

To examine the localization of the transporters in human kidneys at single-cell resolution, we first verified the renal anatomical regions of all single nuclei in the three data sets as described in Methods. The scheme of scRNA-seq analyses and cell clustering were summarized in Additional file 1: Fig. S1A. By unsupervised clustering of the gene count table in the data sets, all single nuclei were divided into 14 clusters. After unsupervised clustering, the 14 clusters were annotated by marker genes (Fig. 1A, Additional file 1: Fig. S1B). The annotation was mainly based on the 9 anatomical regions, and cells in the clusters were calculated as percentages in the regions (Fig. 1B). All cell types of renal tubules were covered by the three analyzed data sets (Additional file 1: Fig. S1C).

Proximal tubule (PT) is the main region, where urate is reabsorbed and secreted, and it is divided into three segments based on anatomical localization. First, *CUBN* and *LRP2* were used as PT-marker genes, and three PT clusters (PTa-c) were annotated (Fig. 1A). Subsequently, using the PT segment-specific marker genes, we were able to designate PTa-c clusters as S1, S2, and S3 segments (Fig. 1C). Thus, the data sets allowed us to evaluate the localization of urate transporters, including three PT segments.

At least eleven genes have been identified as urate transporters in human kidneys (Additional file 1: Fig. S1D) [8–12]. To confirm the transporters and the localization for urate handling in human kidneys, we focused on the expression of eleven known transporters across anatomical regions. Except for *ABCG2*, *ABCC4,* and *SLC22A13*, the remaining eight transporters were specifically expressed in the PT and the descending loop of Henle (DL) (Fig. 1D). *ABCG2* and *SLC22A13* were rarely expressed in all clusters, while *ABCC4* was the only transporter highly expressed in the intercalated cell type A region (Fig. 1D). The results clarified that transport via these eight transporters existed mainly in the PT and to some degree in the DL region. Consequently, our subsequent analyses focused on (1) the four regions (S1, S2, S3, and DL), and (2) the eight urate transporters specifically expressed in the clusters (SLC2A9, SLC17A1, SLC17A3, SLC22A6, SLC22A7, SLC22A8, SLC22A11, and SLC22A12) (Fig. 1E).

The eight transporters have unique characteristics for cellular urate transport. In physiological conditions, the transporters are proposed to be unidirectional based on transport affinity and substrate availability both outside and inside the cells [8, 13]. Table 1 summarizes the eight transporters in terms of their transport systems, substrates, affinities (K_m_) for urate, and average expressions in the clusters of PT and DL [9, 10, 12, 31–38], demonstrating that these transporters can be classified based on their cellular functions for urate transport.

### Classification of four transporter types and four cell populations

To classify the eight transporters, we considered the polarized localization of the transporters (apical or basolateral membranes) and the direction of urate flux (efflux or influx). We categorized the eight transporters into one of the four types (Fig. 2A): Apical Influx transporters (AI transporters), Apical Efflux transporters (AE transporters), Basolateral Influx transporters (BI transporters), and Basolateral Efflux transporters (BE transporters). The cooperation of AI and BE transporters leads renal tubular cells to function as reabsorption, whereas the cooperation of BI and AE transporters leads their cells to function as secretion. This method of classification helped us to predict the cellular urate transport function.

Based on the cellular expression profiles of the transporter types, we hypothesized that cell populations had distinct cellular functions for transport. Based on the gene-positivity of the influx transporters, PT and DL cells were categorized into one of the four cell populations as described in the Methods (Fig. 2B): (1) AI transporter—Positive (AIP) cell population, the cell population expressing influx transporter(s) on the apical membrane but not influx transporter on the basolateral membrane; (2) BI transporter—Positive (BIP) cells population, the cell population expressing influx transporter(s) on the basolateral membrane but not influx transporter on the apical membrane; (3) Dual Influx transporters—Positive (DIP) cell population, the cell population expressing both AI and BI transporters; and (4) Dual Influx transporters—Negative (DIN) cell population, the cell population lacking expressions of both AI and BI transporters. For example, AIP cells expressed *SLC22A11* or *SLC22A12*, or both of them, but did not show expression of *SLC22A6*, *SLC22A7,* or *SLC22A8*. It is noted that the positivity of AI transporter(s) in AIP cells was defined as having the expression of at least one AI transporter (Additional file 1: Fig. S2A), and the positivity of BI transporter(s) in BIP cells was defined as the presence of at least one BI transporter (Additional file 1: Fig. S2B).

To elucidate the expression profiles of the influx transporters in the cell populations, we first examined how frequently the same types of influx transporters were co-expressed. The most frequent AI transporter was *SLC22A11* across the regions (Additional file 1: Fig. S2C, D), while the most frequent BI transporter was *SLC22A6* (Additional file 1: Fig. S2E–G). We found that for AI transporters in all tubular regions, approximately 78–86% of the AIP cell population expressed *SLC22A11* but not *SLC22A12*, and less than 5% of the AIP cell population expressed both *SLC22A11* and *SLC22A12* (Additional file 1: Fig. S2H). By contrast, *SLC22A11*, but not *SLC22A12*, is expressed in approximately 64–81% of the DIP population, and less than 16% of DIP cell population accounted for population with both *SLC22A11* and *SLC22A12* expression (Fig. 2C). For BI transporters, approximately 60% of the BIP cell population in the S1 and S3 regions showed co-expression of two or more different BI transporters, although the proportion decreased in the S2 (33%) and DL (17%) regions (Additional file 1: Fig. S2I). In other words, multiple kinds were co-expressed in BI transporters compared to the AI ones. The BI transporter trend was also found to be common in the DIP cell populations; at least 36% of the DIP cell population expressed two or more different BI transporters (Fig. 2D). These results suggest that only one kind of influx transporter, mostly SLC22A11, is expressed on the apical membrane. By contrast, multiple kinds of influx transporters would be co-expressed on the basolateral membrane, in which SLC22A6 is often found in most cells (Fig. 2E).

### Indispensability of the efflux transporters for prediction of cellular urate transport

**Fig. 3:**
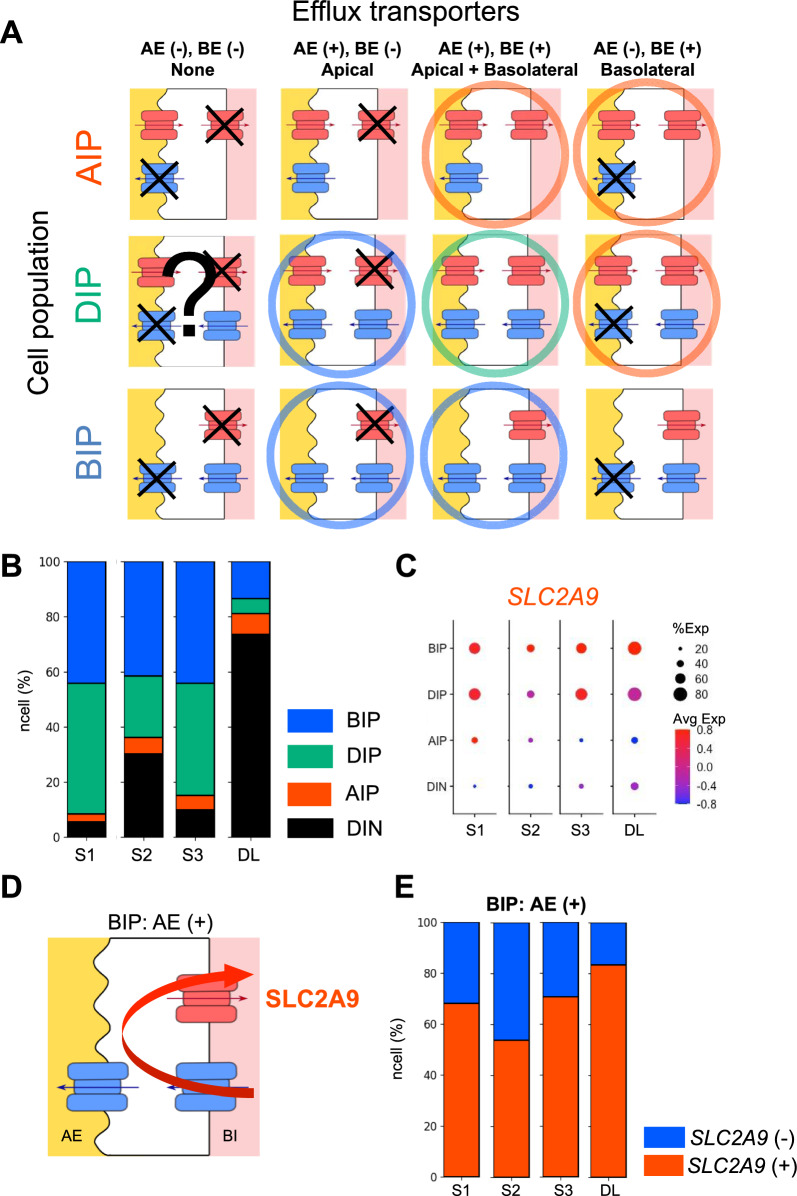
Prediction of cellular urate transport direction from the expression of the transporters. **A** Models indicating all potential expression patterns of urate transporters. Rows indicate cell populations: AIP cell population, BIP cell population, and DIP cell population. Columns indicate expressions of efflux transporters; AE: Apical Efflux transporter; BE: Basolateral Efflux transporters. Colors show the types of transporters: red, AI or BE transporters; blue, BI or AE transporters. Arrows indicate urate transport directions. Functional cells for urate transport are marked with circles in which the circle colors indicate cellular transport directions: red, reabsorption; blue, secretion; and green, bi-directional. **B** Proportion of the cell populations in renal anatomical regions. The bar plot shows the percentage of cells (y-axis) in each cell population across the PT and LOH (DL) clusters (x-axis). **C** Dot plot indicates the frequency and expression levels of *SLC2A9* (x-axis) across the cell populations (y-axis) along the three PT segments (S1–S3) and DL. Dot sizes refer to the frequency of a molecule expressed in the cell population (%Exp), while dot colors indicate expression levels (Avg Exp). **D** Schematic diagram of SLC2A9 expression in the BIP cell population with the Apical Efflux transporters (AE(+)). The apical membrane is to the left of the cells and the basolateral membrane is to the right of the cells. Red symbols represent the AI transporters. Blue symbols represent the BI and AE transporters. Arrows indicate directions of urate flow. **E** Positivity proportion of *SLC2A9* in the BIP cell population with the Apical Efflux transporters. The bar plot shows the percentage of cells (y-axis) in each cell population across the PT and LOH (DL) clusters (x-axis)

We considered transport direction to be key in the prediction of cellular urate flow. Here, we classified the cell populations into three urate transport directions: (1) reabsorption mode, (2) secretion mode, and (3) bidirectional mode. The classifications are based on three criteria. The first criterion is the availability of urate. We assumed that transport direction is based on the availability of urate in both the lumen and the blood. The second criterion is the existence of influx transporters, as the efflux of urate only takes place with the influx of urate into the cells. Accordingly, the DIN cell population was omitted from this analytical step due to a lack of influx transporters (Table 2, Additional file 1: Fig. S3A). Third, we assumed that a complete transport direction mode requires the co-expression of an influx transporter on one side of the polarized membrane (either AI or BI transporter) and an efflux transporter on the other side of the polarized membrane in the same cell. In other words, the AI and BI transporters are assigned as the primitive transporters, while the AE and BE transporters accomplish such transport modes as the derivative transporters. Based on the three criteria, we classified cellular directional modes in the three cell populations: AIP, BIP, and DIP cell populations (Fig. 3A, Table 2). From the analysis, the AIP cell population accommodated reabsorption mode when the AIP cells expressed BE transporter(s) (Fig. 3A: AIP: AE(+), BE(+) or AIP: AE(−), BE( +)). Similarly, the BIP cell population-derived secretion mode when the BIP cells expressed AE transporter(s) (Fig. 3A: BIP: AE(+), BE(+) or BIP: AE(+), BE(−)). In the case of the DIP cell population, reabsorption, secretion, or bidirectional modes can be obtained based on the presence of the types of efflux transporters (Fig. 3A: DIP, Table 2).

**Table 2:** Cellular urate flow based on the localization of the expressed influx (row) and efflux (column) transporters

		Localization of Efflux transporters			
		None (AE(−), BE(−))	Apical (AE( +), BE(−))	Apical + Basolateral (AE(+), BE(+))	Basolateral (AE(−), BE(+))
Localization of Influx Transporters (Cell population)	Apical (AIP)	Unfunctional	Unfunctional	**Reabsorption**	**Reabsorption**
	Apical + Basolateral (DIP)	?	*Secretion*	Bi-direction	**Reabsorption**
	Basolateral (BIP)	Unfunctional	*Secretion*	*Secretion*	Unfunctional
	None (DIN)	Unfunctional	Unfunctional	Unfunctional	Unfunctional

Cellular urate flow consists of three modes: reabsorption (in bold), bi-directional (in underlined), or secretion (in italics)

To visualize urate handling across the renal anatomical regions, we clarified the cell populations that were presented in each region. We found that the BIP and DIP cell populations were highly dominant in the S1 region and gradually decreased along the anatomical regions (Fig. 3B, Additional file 1: Table S4). This suggests that the three cell populations are not only in the PT region but also in the DL region and that their proportion decreases as anatomical localization progressed downward from the S1 region (BIP 44%, DIP 47%) to DL regions (BIP 14%, DIP 5.4%). In all PT segments, the proportion of the BIP cell population was approximately 50%, whereas the AIP cell population (2.9–7.4%) was the smallest among the cell populations (Fig. 3B). These analyses indicated that the cells expressing AI transporters (AIP and DIP cell populations) also expressed BI transporters, but the cells expressing BI transporters (BIP and DIP cell populations) did not necessarily express AI transporters.

To elucidate the expression profiles of the efflux transporters across the cell populations, we analyzed the frequencies and expression levels of the BE and AE transporters (Fig. 3C, Additional file 1: Fig. S3B, C). In all PT and DL clusters, the BIP and DIP cell populations exhibited a high frequency and expression level of *SLC2A9*, the only one known BE transporter, while the AIP cell population showed a low frequency and expression level of *SLC2A9* (Fig. 3C). These results suggest that SLC2A9 is expressed not only in the renal tubular cells related to cellular urate reabsorption (the AIP and DIP cell populations), but also in the renal tubular cells involved in cellular urate secretion (the BIP cell population). The expression of *SLC2A9* in the BIP cell population is interesting, because it suggests the role of SLC2A9 in lowering the secretion efficiency. In other words, the BIP cell population which expressed SLC2A9 (Fig. 3A: BIP: AE(−), BE(+) and BIP: AE(+), BE(+)) can drive urate efflux back to the blood (Fig. 3D). To clarify the regions, where SLC2A9 might decrease secretion efficiency, we analyzed the BIP cell population that expressed *SLC2A9*. The result showed that approximately 70% of the BIP cell population with AE transporter (BIP: AE(+)) expressed *SLC2A9* (68% at S1, 71% at S3, and 83% at DL) (Fig. 3E: red), suggesting that SLC2A9 lowers the secretion efficiency in the BIP cell population, especially at the beginning and the end of urate handling regions (Fig. 3D).

### Contribution of urate transportome to urate handling

**Fig. 4:**
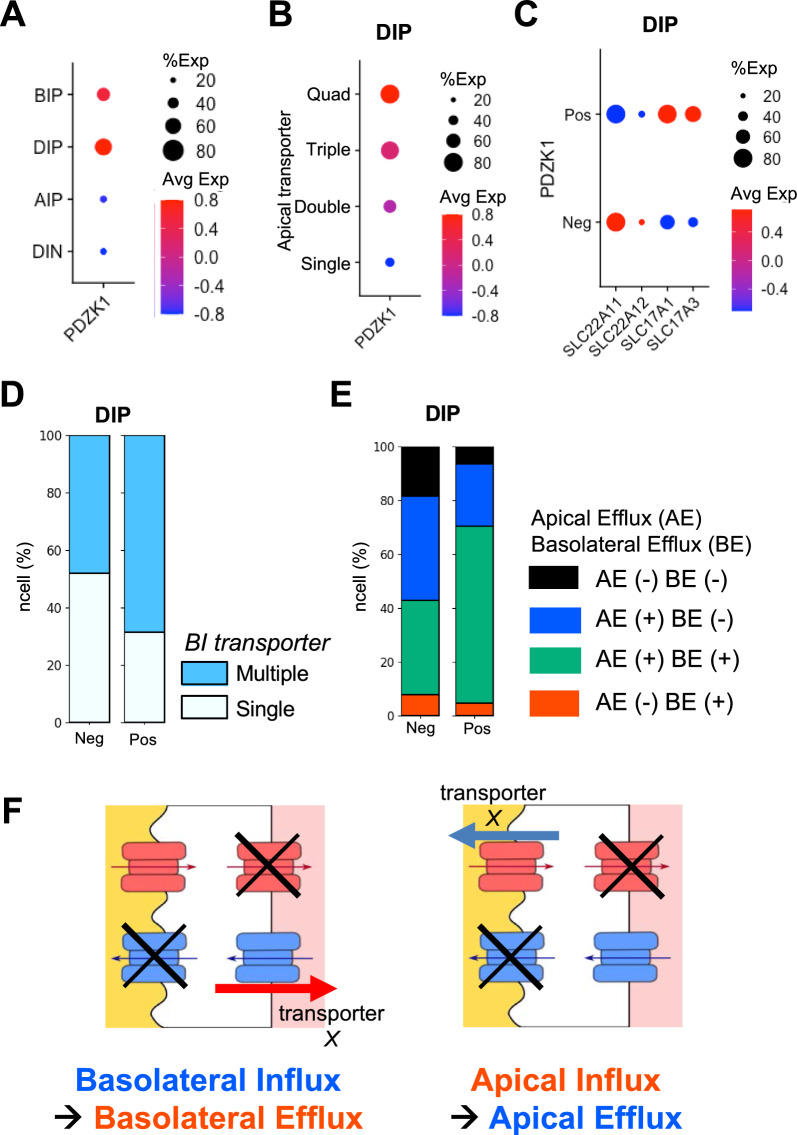
Expression profiles of urate transporters and PDZK1 in DIP cell population. **A** Expression levels and frequencies of *PDZK1* in the cell populations. **B** Expression levels and frequencies of *PDZK1* in the DIP cell population are rearranged by the numbers of apical membrane transporters. **C** Dot plot indicates the frequency and expression levels of the apical transporters (x-axis) across the positivity of *PDZK1* (y-axis). Dot sizes refer to the frequency of a molecule expressed in the cell population (%Exp), while dot colors indicate the average expression levels of clusters (Avg Exp). **D** Proportions of the DIP cell population which express the numbers of BI transporters and *PDZK1*. y-axis, percentage of cells; x-axis, positivity of *PDZK1* (Neg, negative; Pos, positive). **E** Proportions of the DIP cell population which express different types of efflux transporters and *PDZK1*. Y-axis, percentage of cells; x-axis, positivity of *PDZK1* (Neg, negative; Pos, positive). **F** Schematic diagram of transporter reversibility from influx transporters to efflux transporters in the DIP cell population which did not express any efflux transporters. The apical membrane is to the left of the cells and the basolateral membrane is to the right of the cells. Red symbols represent the AI and BE transporters. Blue symbols represent the BI and AE transporters. Arrows indicate directions of urate flow

We considered the presence of some proteins which interact with the transporters and promote urate transport. We hypothesized that one of the key factors is PDZ proteins, which are scaffold proteins that interact with various transmembrane proteins, including transporters, via PDZ motifs at the C-termini. PDZK1 forms the functional urate transport unit (*urate transportome*) by clustering urate transporters (and also other transporters) on the apical membrane [7, 39]. We first investigated the correlation between *PDZK1* expression patterns and cell populations. The results demonstrated that the DIP cell population had the highest frequency and expression level of *PDZK1*. *PDZK1*-positive cells accounted for approximately 60% of the DIP cell population (Fig. 4A), indicating that the DIP cell population expressed *PDZK1* more frequently and strongly than other cell populations.

In contrast to the BIP and AIP cell populations, the DIP cell population potentially directs both secretion and reabsorption and changes the net cellular transport mode by the transporters expressed. To clarify whether the PDZK1-mediated urate *transportome* favors the expression patterns of the transporters, we presented the expression of *PDZK1* over the number of apical transporters. The more diverse the apical urate transporters were, the higher the expression levels and frequencies of *PDZK1* were (Fig. 4B). *PDZK1*-positive cells had higher or equal frequencies of all apical transporters compared to *PDZK1*-negative cells, and the AE transporters (*SLC17A1* and *SLC17A3*) were also more strongly expressed in *PDZK1*-positive cells (Fig. 4C). These results indicate the contribution of PDZK1 to the formation of the cellular urate *transportome* on the apical membranes of the DIP cell population.

To demonstrate the relationship between the positivity of PDZK1 and the net cellular transport mode in the DIP cell population, we focused on the expression of the basolateral transporters in addition to the apical transporters across the positivity of *PDZK1*. As shown in Fig. 2E, the DIP cell population had single or multiple kinds of BI transporters. *PDZK1*-positive cells (68%) more frequently had multiple kinds of BI transporters than *PDZK1*-negative cells (48%) (Fig. 4D). Also, *SLC2A9,* the only AE transporter*,* was expressed more frequently and strongly in *PDZK1*-positive cells (Additional file 1: Fig. S4). Approximately 60% of the *PDZK1*-positive DIP cell population accounted for the positive expressions of both efflux transporter types (AE and BE transporters), while the *PDZK1*-negative DIP cell population more frequently accounted for the negative expressions of the efflux transporter types, including the influx transporter-only population (Fig. 4E). These results suggest that PDZK1 has a positive relationship with basolateral transporters in addition to apical transporters and may facilitate the change of the net cellular transport mode in the DIP cell population.

The fundamental principle of transporters is that the transport direction is determined by concentration gradient of intracellular and extracellular substrates. The concentration ratio is constant with values, which is defined by affinity (Km) of the expressed transporters. From our cell population classification, we observed that efflux transporters were not always expressed in the DIP cell population (Fig. 4E). In fact, approximately 20% of the *PDZK1*-negative DIP cell population expressed none of the efflux transporters (Fig. 4E). It is interesting to examine the cellular urate transport direction of the DIP cell population which did not express efflux transporters, because our observation of cell populations suggest that some influx transporters could switch their transport directions, otherwise, intracellular urate concentration would exceed the equilibrium concentration of an influx transporter. For example, when intracellular urate concentration exceeds the equilibrium concentration of an influx transporter “*X*” on the apical membrane by a basolateral influx transporter (or vice versa), transporter *X* may change to be efflux transporter to lower the intracellular urate concentration to the equilibrium concentration (Fig. 4F). In another word, the DIP cell population without efflux transporters could potentially switch the direction of urate transport from influx to efflux, for example, from an influx transporter to an efflux transporter on the apical or basolateral membrane (Fig. 4F).

### Design of a triple cell-unit model of urate handling, the “*CUTE model*”

**Fig. 5:**
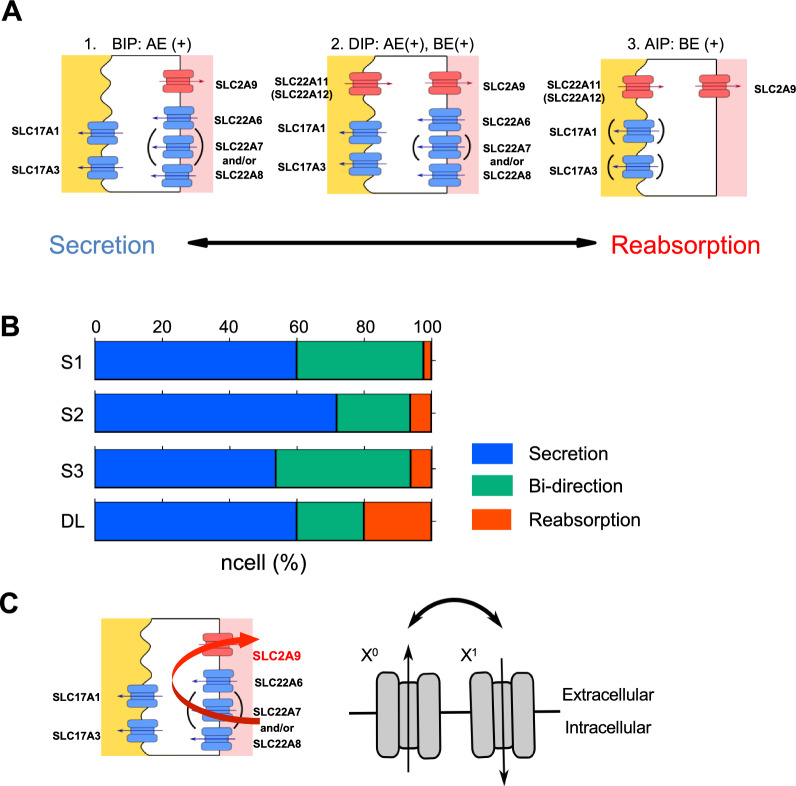
Major cell populations explaining renal urate transport dynamics. **A** *CUTE model* represents cell populations that contributed to urate reabsorption and secretion. Colors indicate types of transporters: red, AI or BE transporters; blue, BI or AE transporters Arrows indicate directions of urate flow. **B** Bar plot showing cell proportion by cellular urate handling in three PT segments and LOH (DL) regions. **C** Schematic diagram illustrating the feasibility of the superiority of urate reabsorption over urate secretion. Left panel: attenuation of urate secretion in BIP cell population by SLC2A9 at all tubular regions. Right panel: reversibility of the transporter function between urate influx and efflux in human kidneys

From the results of three representative cell populations (AIP, BIP, and DIP cell populations), we propose the *Cellular Urate Transport Excretion* (*CUTE*) *model* (Fig. 5A, Additional file 1: Fig. S5A). This framework defines the cellular transport modes (either secretion or reabsorption) from cell populations based on the expression patterns of transporters on apical and basolateral membranes. If the BIP cell population expresses efflux transporters on the apical membrane [Fig. 5A: BIP: AE (+), BE(+) or BIP: AE (+), BE(−)], the population works for urate secretion, which accounted for 1.4% of tubular cells in the PT and DL region. Similarly, if the AIP cell population expresses efflux transporter on the basolateral membrane [Fig. 5A: AIP: AE (+), BE(+) or BIP: AE (−), BE(+)], the population (31% of the tubular cells) works for urate absorption. In the DIP cell population, the cells could work for urate secretion or reabsorption depending on the transporter types expressed (Fig. 5: DIP). The DIP cell population with AE transporter could potentially play a role in urate secretion (Additional file 1: Fig. S5A: DIP: AE (+), BE(−), 9.2% of the tubular cells), and the DIP cell population with BE transporter could participate in urate reabsorption (Additional file 1: Fig. S5A: DIP: AE (−), BE(+), 1.9% of the tubular cells). Meanwhile, DIP populations with none or both apical and basolateral efflux transporters could work bidirectionally (Additional file 1: Fig. S5A: DIP: AE(−) BE(−), 3.5% of the tubular cells or DIP: AE(+) BE(+), 17% of the tubular cells). Our results suggest that not all renal tubular cells express the same set of urate transporters and that the cells involved in the cellular reabsorption are distinct from those involved in the cellular secretion.

To clarify the cell proportion for urate handling across the regions, we quantified the counts for reabsorption cells, bidirectional cells, and secretion cells (Fig. 5B, Additional file 1: Table S4). Proportions of the regions are presented in Additional file 1: Fig. S5B, and the average proportions of the cell populations in the regions are as follows: secretion 63%, bi-directional 32%, and reabsorption 5%. The ratio of reabsorption cells per secretion cells had an upward trend along the renal tubule (S1 0.04, S2 0.09, S3 0.12, and DL 0.33); in particular, the ratio in the DL region was by far the highest among the regions. These results suggest that urate reabsorption is not as superior as urate secretion at the beginning of PT but becomes more significant at S3 and DL. Accordingly, secretion could be most active at the beginning of the PT and gradually decline along the PT segments.

The clarification of the set of urate transporters at single-cell resolution provided some insights into transporter functions. First, SLC2A9 could play a significant role to lower secretion efficiency, which supports the clinical data of low urate excretion (Fig. 5C: left panel). Second, the *CUTE model* proposes the property of transport reversibility in some transporters under physiological conditions (Fig. 5C: right panel). The reversibility of transport direction is not applied to all urate transporters, as found in previous reports. For example, SLC22A7 (a BI transporter) and SLC22A11 (an AI transporter) were reported to be unidirectional urate transporters [32, 35]. In addition, SLC22A12 (an AI transporter) and SLC2A9 (a BE transporter) tend to cooperate in urate reabsorption within co-expressing cells [40], although both transporters possibly divert in opposite directions [9, 33]. Based on these previous findings, the candidate transporters capable of switching their transport directions are the BI transporters SLC22A6 and SLC22A8 (except SLC22A7) and AE transporters SLC17A1 and SLC17A3; AI and BE transporters, which are related to the urate reabsorption, have restricted transport directions. To note, these results were found only when the molecular function on cell units, including both apical and basolateral membrane, was taken into consideration.

## Discussion

The concept of having the same sets of multiple transporter molecules in the same cells has been proposed [8, 13]. Development of scRNA-seq has made the comprehensive quantification of mRNA of transporters in a single cell possible. Our study elucidated these sets of transporters in single cells and predicted the directions of the cellular urate transport. Four factors were used to construct the cellular model. The first factor was the identification of renal anatomical regions, where known urate transporters were expressed; these regions were expected to be the functional regions for reabsorption and secretion. The next factor was the identification of four transporter types, which were defined based on the characteristics of the transporters. The third factor was the classification of four cell populations based on the expression profiles of the transporters at single-cell levels. The combination of the transporter types and the cell populations led to the prediction of cellular direction modes of urate transport as the fourth factor. The *CUTE model* confirmed the expression of the transporter molecules, except SLC22A13, ABCG2, and ABCC4, in human kidneys. In addition, the *CUTE model* clarifies the cellular inhomogeneity (Fig. 5A) and complexity of cell population distribution along the anatomical regions (Fig. 5B). An advantage of having inhomogeneous cell populations is probably the modulation of urate homeostasis. This categorization within the *CUTE model* provides a foundational framework to understand the primary roles these cells play in urate transport at single-cell resolution, resulting in the bridging between molecular functions and physiological excretion processes.

Renal substrate handling by transporters is assumed by specific counterpart between influx and efflux transporters, and each transporter essentially has one fixed cellular function [8–12]. The *CUTE model* proposes a concept that molecular functions of transporters are flexible, and these functions vary, depending on the expression of other transporters in the same cells. One example presented here is the attenuation of urate secretion by SLC2A9 (Fig. 5C: left panel). Another example is the change in cellular transport direction given the reversal property of some transporters in human kidneys (Fig. 5C: right panel). Bidirectional functions of urate transporters in vitro have been previously reported [9, 31, 33, 34]. By contrast, urate transporters in physiological conditions are proposed to be restricted to unidirectional transport based on the transport affinity and substrate availability both outside and inside of the cells [8, 13]. The *CUTE model* suggests that the urate transporters function bidirectionally in vivo as well as in vitro, resulting in changes in cellular transport direction. Further functional analysis by co-expression of apical and basolateral transporters and structural knowledge of difference between reversible and irreversible transporters would help us gain a deeper understanding of the urate cellular handling system.

Knowledge from human samples is essential to truly understanding human physiological systems. The *CUTE model* not only explains the molecular significance of urate transporters but also proposes its clinical relevance. Currently, only two urate transporters related to the Mendelian disorders have been reported: SLC22A12 (OMIM 220150), which is responsible for renal hypouricemia type 1 (RHUC1) [41], and SLC2A9 (OMIM612076) which is responsible for renal hypouricemia type 2 (RHUC2) [42, 43]. Before the characterization of SLC2A9, most of the clinical studies focused on SLC22A12, and over 90% of renal hypouricemia was reported from *SLC22A12* mutations [44]. Here, we suggest the clinical association of SLC2A9, particularly in the elderly male population, because the data sets used in this study were from elderly males. The *CUTE model* advocates SLC2A9 over other transporters because of its single-player BE transporter, high expression profiles, and dual functions in reabsorption and secretion attenuation (Fig. 5C: left panel). Future analysis of more data sets covering a wide range of ages will provide additional clues about other urate transporters. 

The *CUTE model* provides some clues as to where novel urate transporter candidates might be located. Our model suggests the existence of novel transporter(s) involved in urate reabsorption, because the cell number related to reabsorption was lower than the cell number related to secretion (Fig. 5B). A novel BE transporter is a possible candidate; it is likely expressed more frequently and strongly in the AIP and DIP cell populations than in the BIP and DIN cell populations due to the low expression frequency of SLC2A9 in current AIP and DIP cell populations, particularly within the S2 region (Fig. 3B). The model itself provides a ready platform for further analysis when a novel urate transporter is identified, and we believe the analytical model from this study is beneficial for both before and after the discovery of candidate transporters.

Based on the expression of urate transporters at single-cell resolution, the *CUTE model* is constructed to define cellular transport ability. Cellular transport ability is expressed as a vector with orientation and magnitude. However, orientation and magnitude are determined not only by the expression of urate transporters, but also by other factors, including the characteristics of each transporter, such as transport affinity and localization (Table 1). Transport affinity directly affects magnitude of cellular transport ability, such that the total number of cell populations does not necessarily equate to the magnitude of urate cellular transport among the cell populations, even within a cell population. For instance, a cell with a high-affinity transporter for urate may have a higher magnitude of cellular transport than another cell with a lower-affinity transporter. Another important factor is the membrane localization of transporters. We assigned the apical or basolateral localization of all transporters based on the Human Protein Atlas and a thorough review of the existing literature. For all transporters except SLC2A9, there was a consensus in the literature regarding their localization. We treated SLC2A9 as being localized on the apical side due to evidence that pinpointed the localization of human SLC2A9 in the PT region [45], although SLC2A9 may be present on both the apical and basolateral sides [46]. Given that the *CUTE model* is constructed based on the existing and current knowledge, we acknowledge that updates to the model might be required with new and upcoming findings on urate transporters. Nonetheless, we believe that the *CUTE model* would remain relevant as a primary framework.

The series of analyses presented here is highly scalable, and the methodology itself can be a prototype for other metabolite analyses in all organs. It is well-known that multiple transporters in the same cells coordinate to handle a substance across tissue regions, such as nephrons, intestinal epithelia, liver tissues, and blood–brain barrier. Our analytical strategy is useful for broad types of transporters and substrates, leading to an understanding of molecular transport mechanisms in actual physiological systems. Our study suggests the functional cooperation of SLC22A11 and SLC22A6 for urate reabsorption (Fig. 5A). This functional cooperation is also the canonical transport pathway for other endogenous anion substrates or drugs [47–49]. The methodology employed in this study is potentially a strong tool for the elucidation of the transport mechanism in physiological and pathological systems.

In interpreting the results, it is important to understand the limitations of the scRNA-seq data we used. We did not consider factors, such as race/ethnicity, sex, and age, although they have been reported to impact serum uric acid levels [50, 51]. These three factors may cause different expression levels and/or frequencies of urate transporters, as seen in the case of SLC22A12 [52]. Recently, SLC22A13 was found to contribute to urate reabsorption in an elderly Japanese male [53]. In this study, expression of SLC22A13 was not detected (Fig. 1D). It is possible that race is a factor in this variation because of the non-Asian scRNA-seq data we used in this study. As sequencing technology advances, future studies, including metadata analyses, are expected to address the importance of these factors. The *CUTE model* can then be a prototype of healthy male human data sets and be applied to the analyses of pathological conditions, sex differences, and interspecies variation.

## Conclusions

We analyzed renal urate handling at single-cell resolution and proposed the *CUTE model*. The *CUTE model* presents three representative cell populations with distinct transporter expression patterns and the differential distribution of the cell populations along the tubular regions. The construction of the model clarifies that the molecule function of the transporters can be varied and differs across the expression patterns of transporters on single-cell units. Urate excretion was regulated by (1) reabsorption cells (5% of the cell population) which definitely drive urate reabsorption; (2) secretion cells (63%) which are supposed to drive urate secretion, but whose process could be attenuated by high SLC2A9 affinity; and (3) bidirectional cells (32%) with potentially both reabsorption and secretion functions, but tentatively drive the reabsorption over the secretion by the reversibility of some transporters. The inhomogeneity of the cell populations allowed us to predict distinct net cellular transport modes, suggesting that the cells for urate reabsorption are relatively different from the cells for urate secretion. The observed distribution indicates that the proximal tubules and, to some degree, the descending loop of Henle, are the most active regions for urate transport, which leads us to predict the urate excretion dynamics in certain regions of the nephrons. Overall, the urate transport dynamics described by our model indicate the superiority of urate reabsorption over urate secretion, leading to low uric acid excretion. Our methodology to provide the cellular transport direction reflects the basic concept that any process, whether secretion or reabsorption, must pass through at least two types of transporters: one on the apical membrane and the other on the basolateral membrane. We believe our approach can be applied to the analysis of transporter systems in general, and help bridge the gap from molecular function to cellular function.

### Supplementary Information

**Additional file 1: Figure S1.** Identification of renal cell types in the data sets. (A) Analysis flowchart of scRNA-seq. (B) Unsupervised clustering of healthy human adult renal cells after the annotation. A UMAP plot visualizes cell similarity of gene expression across to make cell clusters of similar gene expressions. The clusters were named after the anatomical regions of the kidneys: Podo, podocyte; PTa-c, three clusters of proximal tubules; LOH (DL), the loop of Henle (descending loop); LOH (AL), the loop of Henle (ascending loop); DCT, distal convoluted tubule; CNT, connecting tubule; PC, principal cell; ICA, intercalated cell type A; ICB, intercalated cell type B; EDC, endothelial cell; MGC, mesangial cell; and PEC, parietal epithelial cell. (C) Unsupervised clustering of the three human data sets. (D) Schematic diagram of the subcellular localization of eleven known urate transporters. The apical membrane is to the left of the cells, and the basolateral membrane is to the right of the cells. Red symbols represent transporters which are reconstituted to urate reabsorption. Blue symbols represent transporters which are reconstituted to urate secretion. Arrows indicate directions of urate flow. **Figure S2**. Expression of influx transporters in each cluster. (A) Schematic indicating the positivity of AI transporters. The apical membrane is to the left of the cells, and the basolateral membrane is to the right of the cells. Red symbols represent transporters which are reconstituted to urate reabsorption. Arrows indicate directions of urate flow. (B) Schematic indicating the positivity of BI transporters. Blue symbols represent transporters which are reconstituted to urate secretion. (C–G) Dot plots indicate the frequency and expression levels of urate transporters (x-axis) across the cell populations (y-axis) along the three PT segments (S1–S3) and DL. Dot sizes refer to the frequency of a molecule expressed in the cell population (%Exp), while dot colors indicate the expression levels (Avg Exp). *SLC22A11*(C) and *SLC22A12* (D) are AI transporters described in red letters. *SLC22A6* (E), *SLC22A7* (F), and *SLC22A8* (G) are BI transporters described in blue letters. (H, I) Bar plots indicate the percentage (y-axis) of AI transporters in the AIP cell population (H) and BI transporters in the BIP cell population (I) across the regions (x-axis). **Figure S3.** Expression of efflux transporters in each cluster. (A) Models illustrate the potential expression patterns of urate transporters in the DIN cell population. Colors indicate types of transporters: red, BE transporters; blue, AE transporters. Arrows show urate transport directions. (B, C) Dot plots indicate the frequency and the expression levels of the AE transporters (B: *SLC17A1*, C: *SLC17A3*) (x-axis) across the cell populations (y-axis) along the three PT segments (S1–S3) and DL. Dot sizes refer to the frequency of a molecule expressed in the cell population (%Exp), while dot colors indicate the expression levels (Avg Exp). **Figure S4.** Relationship of basolateral transporters and PDZK1. Dot plot indicates the frequency and the expression levels of the basolateral transporters (x-axis) across the positivity of *PDZK1* (y-axis). Dot sizes refer to the frequency of a molecule expressed in the cell population (%Exp), while dot colors indicate the expression levels (Avg Exp). **Figure S5.** Schematic models of urate transport modes in each cell population. (A) Representative cell populations contributed to urate reabsorption and secretion in the DIP cell population. Colors indicate types of transporters: red, AI or BE transporters; blue, BI or AE transporters. (B) Proportions of cell clusters in three PT segments and LOH (DL) clusters in the data sets. PT_S1, S1 segment of proximal tubule; PT_S2, S2 segment of proximal tubule; PT_S3, S3 segment of proximal tubule; and LOH (DL), the loop of Henle (descending loop). **Table S1.** Information of snRNA-seq data sets analyzed in this study. **Table S2.** Marker genes and cell numbers in each renal tubular regional cluster. **Table S3.** Marker genes and cell numbers in three segments (S1–S3) of proximal tubule (PT) clusters. **Table S4.** Analyses of cell populations in the *Cellular Urate Transport Excretion model****.*** Column colors in “Urate transport mode” indicates the role in cellular urate handling; blue (secretion), green (bi-direction), red (reabsorption), gray (nonfunction).

## Data Availability

R and Python codes for data analysis and visualization is available in GitHub repository at https://github.com/yoshi-sci/urate-transporter-analysis.
