# Supplementary material for: Identification of three distinct cell populations for urate excretion in human kidneys
Source: J Physiol Sci. 2024 Jan 2;74:1. doi: 10.1186/s12576-023-00894-0 (PMC10763458; doi:10.1186/s12576-023-00894-0)
Supplement: Supplementary file 1 — Additional file 1: Figure S1. Identification of renal cell types in the data sets. (A) Analysis flowchart of scRNA-seq. (B) Unsupervised clustering of healthy human adult renal cells after the annotation. A UMAP plot visualizes cell similarity of gene expression across to make cell clusters of similar gene expressions. The clusters were named after the anatomical regions of the kidneys: Podo, podocyte; PTa-c, three clusters of proximal tubules; LOH (DL), the loop of Henle (descending loop); LOH (AL), the loop of Henle (ascending loop); DCT, distal convoluted tubule; CNT, connecting tubule; PC, principal cell; ICA, intercalated cell type A; ICB, intercalated cell type B; EDC, endothelial cell; MGC, mesangial cell; and PEC, parietal epithelial cell. (C) Unsupervised clustering of the three human data sets. (D) Schematic diagram of the subcellular localization of eleven known urate transporters. The apical membrane is to the left of the cells, and the basolateral membrane is to the right of the cells. Red symbols represent transporters which are reconstituted to urate reabsorption. Blue symbols represent transporters which are reconstituted to urate secretion. Arrows indicate directions of urate flow. Figure S2. Expression of influx transporters in each cluster. (A) Schematic indicating the positivity of AI transporters. The apical membrane is to the left of the cells, and the basolateral membrane is to the right of the cells. Red symbols represent transporters which are reconstituted to urate reabsorption. Arrows indicate directions of urate flow. (B) Schematic indicating the positivity of BI transporters. Blue symbols represent transporters which are reconstituted to urate secretion. (C–G) Dot plots indicate the frequency and expression levels of urate transporters (x-axis) across the cell populations (y-axis) along the three PT segments (S1–S3) and DL. Dot sizes refer to the frequency of a molecule expressed in the cell population (%Exp), while dot colors indicate t [file 12576_2023_894_MOESM1_ESM.docx]

Additional file

# Additional Figures and Tables

## Additional Figures

##

**Figure S1. Identification of renal cell types in the datasets.** (A) Analysis flowchart of scRNA-seq. (B) Unsupervised clustering of healthy human adult renal cells after the annotation. A UMAP plot visualizes cell similarity of gene expression across to make cell clusters of similar gene expressions. The clusters were named after the anatomical regions of the kidneys: Podo, podocyte; PTa - c, three clusters of proximal tubules; LOH (DL), the loop of Henle (descending loop); LOH (AL), the loop of Henle (ascending loop); DCT, distal convoluted tubule; CNT, connecting tubule; PC, principal cell; ICA, intercalated cell type A; ICB, intercalated cell type B; EDC, endothelial cell; MGC, mesangial cell; and PEC, parietal epithelial cell. (C) Unsupervised clustering of the three human datasets. (D) Schematic diagram of the subcellular localization of eleven known urate transporters. The apical membrane is to the left of the cells, and the basolateral membrane is to the right of the cells. Red symbols represent transporters which are reconstituted to urate reabsorption. Blue symbols represent transporters which are reconstituted to urate secretion. Arrows indicate directions of urate flow.

**Figure S2.** **Expression of influx transporters in each cluster.** (A) Schematic indicating the positivity of AI transporters. The apical membrane is to the left of the cells, and the basolateral membrane is to the right of the cells. Red symbols represent transporters which are reconstituted to urate reabsorption. Arrows indicate directions of urate flow. (B) Schematic indicating the positivity of BI transporters. Blue symbols represent transporters which are reconstituted to urate secretion. (C – G) Dot plots indicate the frequency and expression levels of urate transporters (x-axis) across the cell populations (y-axis) along the three PT segments (S1 - S3) and DL. Dot sizes refer to the frequency of a molecule expressed in the cell population (%Exp), while dot colors indicate the expression levels (Avg Exp). *SLC22A11*(C) and *SLC22A12* (D) are AI transporters described in red letters. *SLC22A6* (E), *SLC22A7* (F), and *SLC22A8* (G) are BI transporters described in blue letters. (H, I) Bar plots indicate the percentage (y-axis) of AI transporters in the AIP cell population (H) and BI transporters in the BIP cell population (I) across the regions (x-axis).

**Figure S3. Expression of efflux transporters in each cluster.** (A) Models illustrate the potential expression patterns of urate transporters in the DIN cell population. Colors indicate types of transporters: red, BE transporters; blue, AE transporters. Arrows show urate transport directions. (B, C) Dot plots indicate the frequency and the expression levels of the AE transporters (B: *SLC17A1*, C: *SLC17A3*) (x-axis) across the cell populations (y-axis) along the three PT segments (S1 - S3) and DL. Dot sizes refer to the frequency of a molecule expressed in the cell population (%Exp), while dot colors indicate the expression levels (Avg Exp).

**Figure S4. Relationship of basolateral transporters and PDZK1.** Dot plot indicates the frequency and the expression levels of the basolateral transporters (x-axis) across the positivity of *PDZK1* (y-axis). Dot sizes refer to the frequency of a molecule expressed in the cell population (%Exp), while dot colors indicate the expression levels (Avg Exp).

**Figure S5. Schematic models of urate transport modes in each cell population.** (A) Representative cell populations contributed to urate reabsorption and secretion in the DIP cell population. Colors indicate types of transporters: red, AI or BE transporters; blue, BI or AE transporters. (B) Proportions of cell clusters in three PT segments and LOH (DL) clusters in the datasets. PT_S1, S1 segment of proximal tubule; PT_S2, S2 segment of proximal tubule; PT_S3, S3 segment of proximal tubule; and LOH (DL), the loop of Henle (descending loop).

## Additional Tables

**Table S1.** **Information of snRNA-seq datasets analyzed in this study.**

**Table S2.** **Marker genes and cell numbers in each renal tubular regional cluster.**

**Table S3. Marker genes and cell numbers in three segments (S1 – S3) of proximal tubule (PT) clusters.**

**Table S4.** Analyses of cell populations in the *Cellular Urate Transport Excretion model.* Column colors in “Urate transport mode” indicates the role in cellular urate handling; blue (secretion), green (bi-direction), red (reabsorption), gray (nonfunction).
